# Cone beam CT angiography-guided direct puncture embolization for treatment of vascular anomalies in the head and neck region

**DOI:** 10.1259/bjrcr.20210226

**Published:** 2022-04-25

**Authors:** Prijo Sidipratomo, Jacub Pandelaki, Heltara Ramandika, Theddeus OH Prasetyono, Jongmin Lee

**Affiliations:** 1 Department of Radiology, Dr. Cipto Mangunkusumo Hospital / Faculty of Medicine Universitas Indonesia, Jakarta, Indonesia; 2 Division of Plastic Surgery, Department of Surgery, Dr. Cipto Mangunkusumo Hospital / Faculty of Medicine Universitas Indonesia, Jakarta, Indonesia; 3 Department of Radiology, Kyungpook National University Hospital, Daegu, South Korea

## Abstract

The use of angiography combined with cone-beam computed tomography (CBCT) in interventional radiology and endovascular surgery is beneficial. The combination of CBCT with digital subtraction angiography resulted in a detailed vascular map and its surrounding structures. This paper presents cases of vascular anomalies outside the skull, including malformations and hypervascular tumors, specifically in the head and neck region, which were managed with direct puncture embolization under CBCT guidance. CBCT could facilitate the visualization and identification of the precise puncture site of targeting vessels. No complications were observed in all cases.

## Summary

The goal of embolization in vascular anomalies is nidus occlusion or regression.^1^ On the other hand, the goal of embolization in hypervascular tumors is to limit blood loss during surgery and reduce surgery duration.^2^ However, in cases of vascular anomalies and hypervascular tumor in the head and neck region, endovascular embolization is technically difficult and might result in an incomplete seal. It may also result in the failure of microcatheter insertion into the targeted vessels, especially in a complex vascular network and smaller vessel caliber than the microcatheter. Therefore, directly puncturing the targeted vessels adjacent to the nidus or feeding artery is preferred.^3^

A combination of angiography and cone-beam computed tomography (CBCT) in interventional procedures, and endovascular surgery is beneficial. It eliminates the need to mobilize patients into different planes. On the other hand, CBCT could provide multiplanar views, including coronal, sagittal, axial, and oblique views. The combination of digital subtraction angiography (DSA) and CBCT results in a detailed picture of the vascular network (feeding artery, nidus, draining vein) and superficial structures of vascular lesion (soft tissue and bones).^4^ The combination also allows precise direct puncture to the targeted vessels.

This study aims to describe our experiences with CBCT-angiography (CBCT-A) guided direct puncture embolization in cases of vascular anomalies and hypervascular tumor in the head and neck region.

## Case description

This study includes patients who were diagnosed with vascular anomalies or hypervascular tumors in the head and neck region and received an embolization procedure, either as a definitive or pre-operative treatment, from January to December 2018 in a single tertiary hospital. A 1-year period was chosen because of the availability of the subjects who had satisfied the inclusion criteria and been followed up for 3 years. We reviewed clinical and angiographic data (age, gender, symptoms, procedure details, and outcomes) from medical records, pre-procedural imaging data (ultrasound, CT, or MRI) and imaging data during the procedure (CBCT-A and DSA images).

There were three patients with vascular malformation and only one patient with vascular tumor. The diagnosis was established based on ultrasound, CT, and/or MRI and later confirmed by DSA.

Superselective angiography was performed using 3D-DSA to identify feeding arteries or vessels adjacent to the nidus in the vascular anomaly. CBCT (Syngo DynaCT; Siemens, Erlangen, Germany) was performed following DSA to obtain information regarding vascular lesion structures. Images acquired from both modalities were combined and reconstructed to create a multiplanar image with a detailed look at the architecture of vascular lesion. A 22G spinal needle (Spinocan; B Braun, Melsungen, Germany) was introduced to the target vessel under the guidance of virtual needle projection on reconstructed images of CBCT-A. The study mixed Histoacryl (N-butyl cyanoacrylate; B Braun, Melsungen, Germany) with Lipiodol (ethiodol; Guerbet, Roissy, France) in the ratio of 1:4 and then administered to the target vessel. Other embolic materials, such as ethanol, were not used in this study. A successful embolization was defined as complete occlusion of nidus and feeding arteries in vascular anomalies or absence of hypervascular appearance in tumors. There were no difficulties during puncture procedure regarding skin deviation.

### Case 1

A 25-year-old female presented with a growing mass on the right forehead accompanied by headache and pain of the right eye in the past year. Ultrasound examination revealed a highly vascularized solid mass with a nidus component on the right frontal region. Color and spectral Doppler on some of the vascular components showed feeding artery with low resistance and high velocities, while draining vein demonstrated throbbing venous flow, signifying the shunt in the arteries and veins. CT and CT angiography (CTA) revealed a serpiginous lesion forming nidus at the frontal subcutaneous region, suggesting an arteriovenous malformation (AVM) with feeding vessel from the right ophthalmic artery. However, DSA revealed two niduses, one at the frontal region with a feeding artery from the right superficial temporal artery and another one confirmed by the CTA. The draining veins were the right temporal, right superior ophthalmic, and bilateral facial veins.

**Figure 1.**
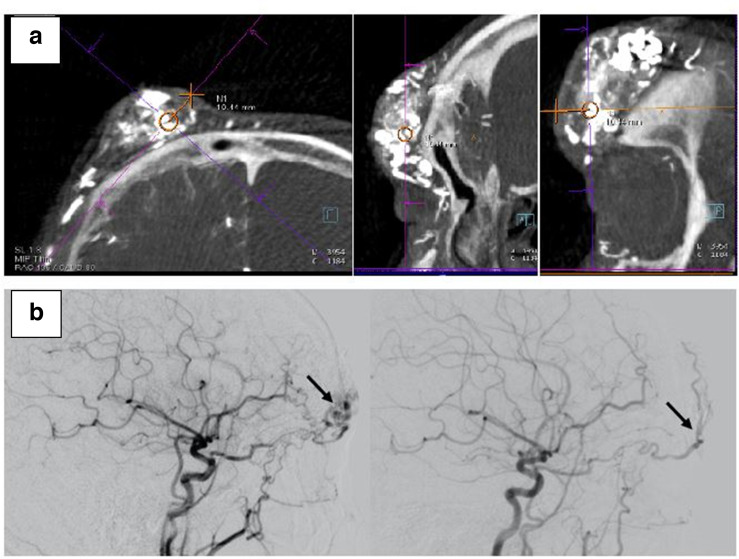
Case 1, AVM of the right frontal region. (a) CBCT-A in axial, sagittal, and coronal views demonstrating the projection of the virtual needle targeting the right supratrochlear artery. (b) DSA images showing (left) nidus at the left frontal region and (right) nidus occlusion following glue administration. AVM, arteriovenous malformation; CBCT-A, cone beam computed tomography angiography; DSA, digital subtraction angiography

Direct puncture embolization targeted the right superficial temporal artery, which successfully obliterated the frontal nidus. Considering the risk of occluding the right ophthalmic artery, we utilized CBCT-A in the second procedure of direct puncture embolization to target the right supratrochlear artery that lay adjacent to the AVM. Complete nidus occlusion was achieved without any complication (Figure 1).

### Case 2

A 23-year-old female presented with a slow-growing mass on the left forehead extending to the upper eyelid since birth. The mass occasionally bled, especially during hot summer. The ultrasound revealed high flow vascular malformation with the nidus component of the left hemifacial, predominantly on the left frontal region. Feeding arteries demonstrate arterial flow with low resistance and high velocity while draining veins demonstrate pulsatile venous flow. These findings imply that arteriovenous shunt exists within the malformation. CTA revealed a serpiginous lesion forming nidus at the bilateral frontal region, left parietotemporal region, left palpebra, and left upper jaw region, suggesting an AVM. DSA confirmed the diagnosis and identified multiple feeding arteries from bilateral ophthalmic and superficial temporal arteries and the left facial, internal maxilla, middle meningeal, and accessory meningeal artery. The draining veins were bilateral ophthalmic, facial, and temporal veins. Embolization was performed and followed by surgical resection and reconstruction.

**Figure 2.**
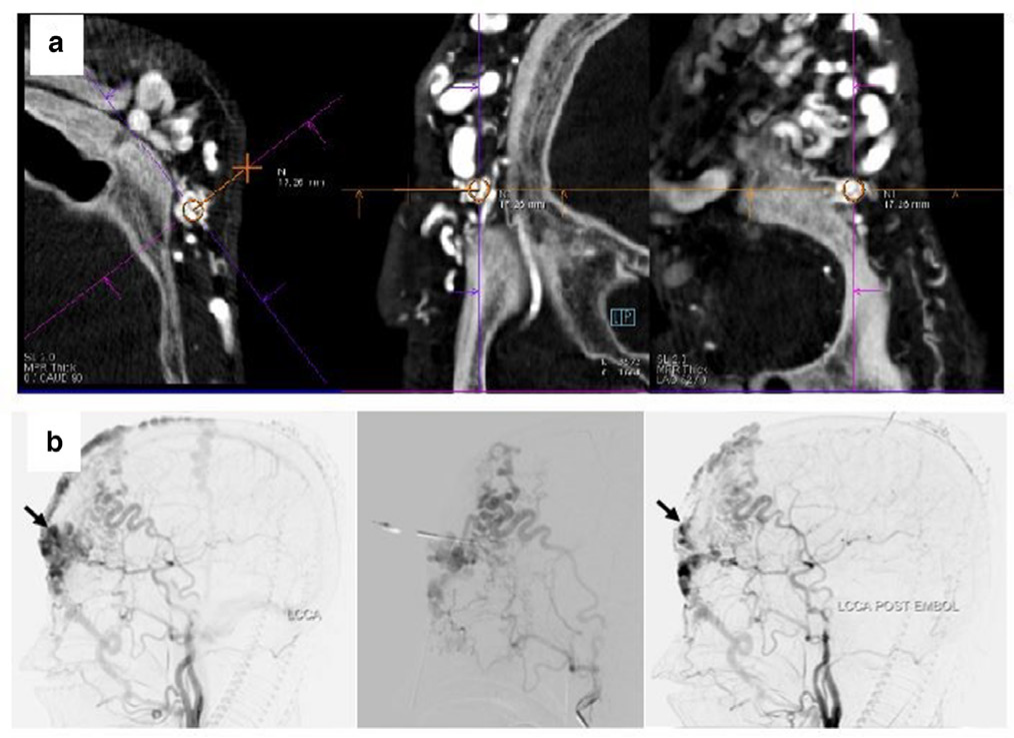
Case 2, AVM of the left frontotemporoparietal and superior palpebra. (a) CBCT-A in axial, sagittal, and coronal views demonstrating the projection of the virtual needle targeting the left superficial temporal artery. (b) DSA images showing (left) multiple nidus at the left frontotemporoparietal dan superior palpebra region, (middle) needle insertion to the target vessel, and (right) nidus occlusion at the left frontal region following glue administration. AVM, arteriovenous malformation; CBCT-A, cone beam computed tomography angiography; DSA, digital subtraction angiography.

Initial embolization procedure inside the blood vessels targeting the main feeding artery, namely the left superficial temporal artery, failed because of the vascular length and torsion. Therefore, we performed direct puncture embolization under the guidance of CBCT-A, which succeeded in occluding the left superficial temporal and internal maxillary artery (Figure 2). No complications occurred. The surgery successfully removed about a quarter of the mass without the necessity of blood transfusion. Due to technical reasons, mass removal was decided as staged surgical interventions. CTA examination performed one month after the surgery revealed a dural fistula of the left frontal to superior palpebral region, supplied by the left middle meningeal and accessory meningeal artery. The patient had not decided whether to undergo another session of embolization and surgical resection when the report was prepared.

### Case 3

A 14-year-old female presented with a mass on the left eyelid since birth. The mass exponentially grew when she was 3 years and occasionally bled in the past 8 years. Physical examination revealed no light perception on the left eye, while MRI revealed a heterogeneous mass with multiple microcysts on the frontotemporal–orbital–zygomatic region. The mass demonstrated heterogeneous enhancement, suggesting a lymphovenous malformation. Despite the low flow characteristic of the malformation, subsequent DSA revealed prominent hyperplastic arteries from the left ophthalmic artery supplying the malformation. Based on DSA, the examiner identified prominent dilated and tortuous veins on the left frontotemporal region. Considering the mass size and the fact that the left eye has no more light perception, we performed excision of the mass and enucleation of the left eye, proceeded by pre-operative embolization.

**Figure 3.**
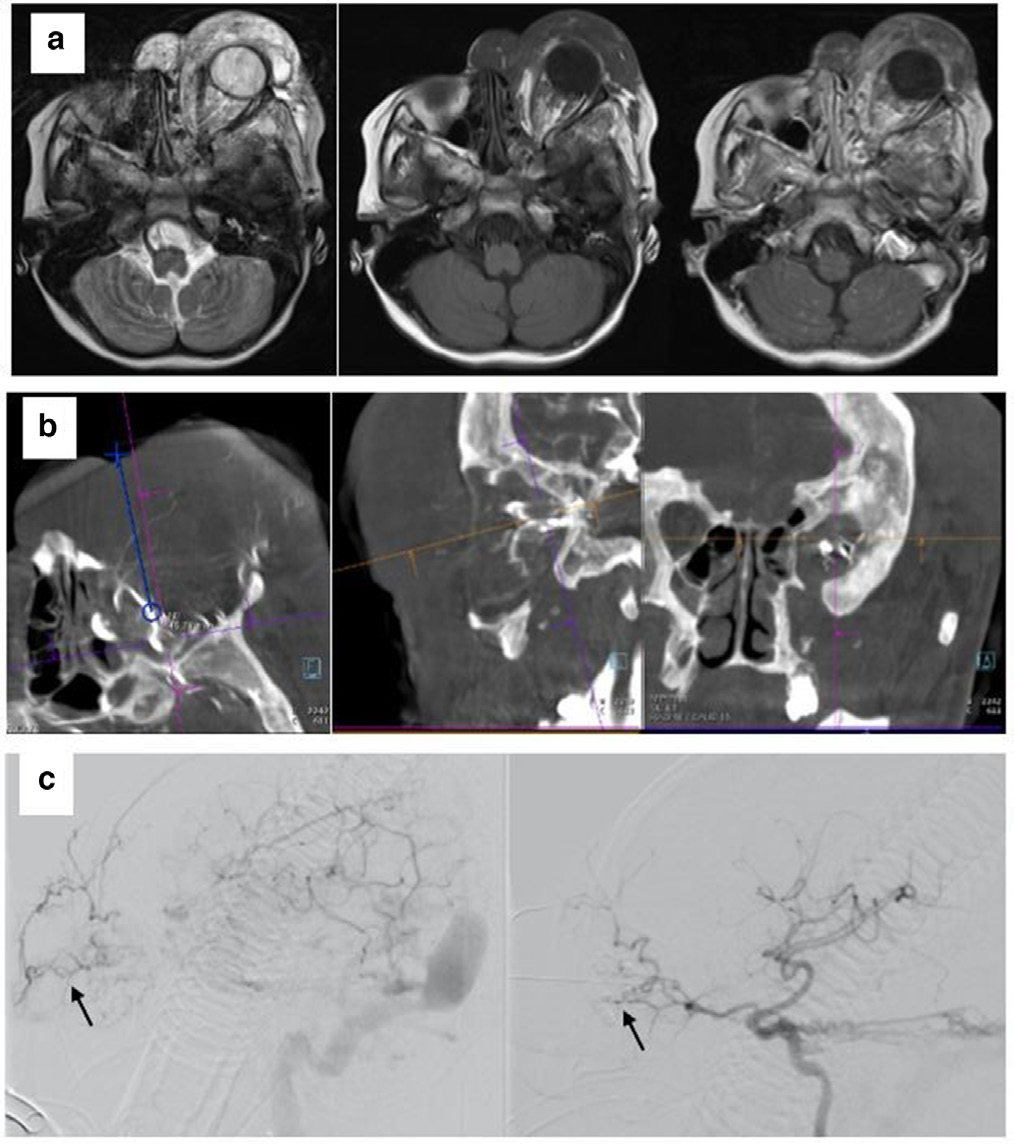
Case 3, Lymphovenous malformation of the frontotemporoorbitozygoma region. (a) Contrast-enhanced MRI revealing a microcystic mass of the left frontotemporo-orbital region with heterogeneous enhancement. (b) CBCT-A in axial, sagittal, and coronal views demonstrating the projection of the virtual needle targeting the left ophthalmic artery. (c) DSA images showing (left) feeding artery, namely the left ophthalmic artery, (right) needle insertion to the target vessel, and occlusion of the feeding artery following glue administration. CBCT-A, cone beam computed tomography angiography; DSA, digital subtraction angiography.

Initially, the endovascular embolization procedure to occlude the left ophthalmic artery failed due to the small diameter of the left ophthalmic artery. Therefore, we performed CBCT-A guided direct puncture embolization. Occlusion of the feeding artery was achieved without any complication (Figure 3). Excisional surgery and reconstruction were uneventful.

### Case 4

A 41-year-old male appeared with a mass on the right eyelid. He suffered intense headaches and frequent bleeding for the past 2 years. Physical examination revealed no light perception on the right eye. MRI showed a right retrobulbar mass with tubular flow void structure inside the mass, suggesting a hypervascular tumor, causing proptosis. Likewise, CTA revealed hypervascular retrobulbar tumor supplied by hyperplastic superior and inferior branches of the right ophthalmic artery. Embolization was conducted, followed by mass resection and exenteration of the right orbit.

**Figure 4.**
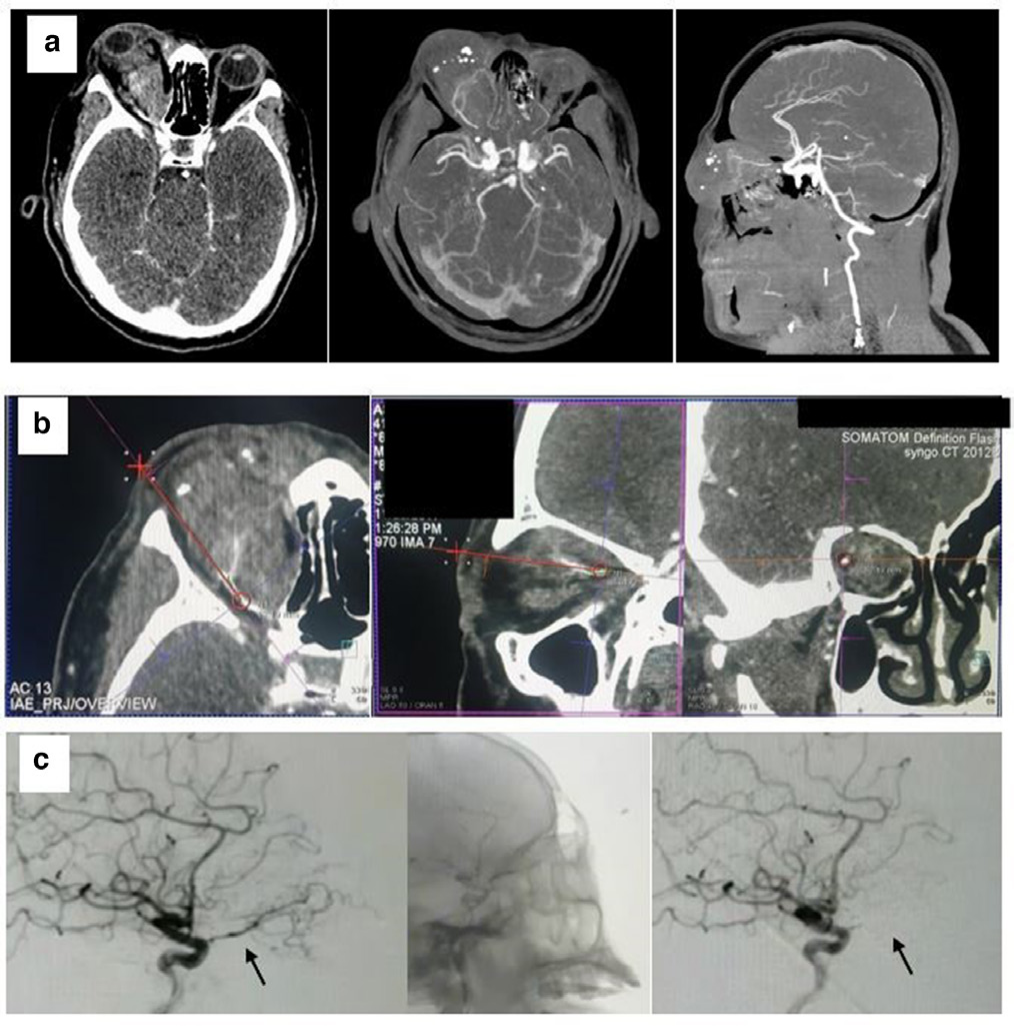
Case 4, hypervascular tumor of the right retroorbital region. (a) CTA of the carotid showing the hypervascular mass of the right retroorbital region supplied by branches of the right ophthalmic artery. (b) CBCT-A in axial, sagittal, and coronal views demonstrating the projection of the virtual needle targeting the right ophthalmic artery. (c) DSA images showing (left) feeding artery, namely the right ophthalmic artery, (middle) needle insertion to the target vessel, and (right) occlusion of the feeding artery following glue administration. CBCT-A, cone beam computed tomography angiography; CTA, computed tomography angiography; DSA, digital subtraction angiography.

Endovascular embolization was planned to target the right ophthalmic artery. However, the tortuosity of the internal carotid artery and ophthalmic artery makes selective catheterization difficult to achieve. A CBCT-A guided direct puncture embolization was performed. The feeding artery was wholly occluded uneventfully (Figure 4). The following excisional surgery and reconstruction were successful. Histopathology result from pathological anatomy examination was vascular malformation. Specific staining tests such as immunohistochemistry examination were not conducted to identify the type of malformation.

## Discussion

Treatment options for vascular anomalies and hypervascular tumors are varied. It is subject to the individual case besides resource availability and is influenced by its value. The options range from minimally invasive approaches to surgical procedures. Embolization might be used either as pre-operative or definitive treatment.^5^ Direct puncture embolization is preferred over the endovascular approach in distal, peripheral, or superficial lesion, distant from femoral artery access, as it provides more efficient access to the dominant feeding arteries.^3^ Considering its ability to create a detailed vascular map, CBCT-A can direct the operators to the target arteries precisely, increasing patient safety and is timesaving.^4^ A study by Nesbit et al^6^ reported that approximately 95% (62/65) of target vessels were successfully punctured directly using CBCT guidance alone. DSA can complement the CBCT in providing puncture guidance to achieve the target vessel. The utilization of DSA will surely increase the accuracy and success rate of direct puncture embolization.

The first step of CBCT procedure is to position the patient so that the target area is within the estimated scan volume. Careful planning is required due to the restricted scopes of the detector. Therefore, position the patient so that the C-arm can rotate around the patient smoothly is important. There are two basic C-arm positions that allow for obtaining a CT cone-beam volume, which are the patient’s head position (propeller scan, usually 240⁰ rotation) and the tableside C-arm (roll scan, usually 180⁰).^7^ A large number of projections can help to reduce artifacts as much as possible. Then, better image quality is obtained by performing scans with as many rotations as possible. Generally, propeller scans are faster in acquisition resulting in better image quality, depending on the characteristics of the angiosystem.

After acquisition, the CBCT volume was reconstructed, and with dedicated needle guidance navigation software, a virtual needle path could be drawn within the volume. Then, by connecting planned virtual 3D path on C-arm with special software, real-time feedback between the C-arm movement and the virtual planning is delivered. It enables automatic positioning of the C-arm at the precise desired angle for needle guidance based on the pre-meditated path. Additional volume acquisition may be performed during the procedure after puncture in order to verify needle position gradually as well as possible complications, such as bleeding and pneumothorax.^7^

The effective radiation dose for patients with CBCT guidance is between 7.6 and 16.1 mSv, depends on the anatomic category. Extensive dose measurements in patients using the CBCT technique compared to conventional CT showed a significant reduction in radiation dose. The dose reduction is between 13 and 43%, depending on the anatomical position.^7^ It is possible to consistently reduce further dose by using horizontal collimation, as it scopes at a smaller volume, only the area to be examined. The total fluoroscopy time with this procedure is limited and rarely exceeds 3 min.

The combination of CBCT with DSA provides multiplanar views of vascular and anatomical structures, which helped the operator embolize the lesion. In one of the cases we reported, CBCT-A helped the operator target the feeding artery closer to the nidus to protect the ophthalmic artery, preserving the visual function. In the other cases, CBCT-A facilitated the success of dealing with the anatomical problems, including vascular length, tortuosity, and small diameter of the target vessels.

CBCT-A could be used either as an adjunctive procedure before the surgical intervention or definitive treatment, as shown in one case. Three cases resulted in complete occlusion of the nidus or feeding artery, while one case resulted in partial occlusion of feeding arteries due to large numbers of feeding arteries. DSA could provide a clear view of blood vessels without interfering with shadows from overlapping tissues. Combination of CBCT and DSA provides visual aid of feeding artery, nidus, draining vein, and the soft tissue structure around it, allowing the operators to perform precise direct percutaneous puncture. Nevertheless, this report highlights the benefit of using direct puncture embolization of CBCT-A.

The success of direct puncture embolization can be evaluated both radiologically and clinically. DSA reveals the success of embolization, which subsequently facilitates the surgery with excision of the mass that can be performed successfully with minimal intraoperative bleeding. Although pre-operative embolization in one case of vascular anomaly resulted in partial occlusion of the nidus, surgery could be executed without significant intraoperative hemorrhage. Overall, pre-operative embolization managed to reduce vascularization of the lesions.

There are several limitations about the application of CBCT. Compared to conventional CT, volume CT CBCT detectors have lower contrast resolution (varies from 5 to 20 HU).^7^ It is also more prone towards artifact formation such as motion (due to slower rotation), noise, and beam hardening due to cone-beam acquisition. CBCT also has limitation when applied on upper extremities due to C-arm difficulties in rotating around it.

In summary, CBCT-A guided direct puncture embolization is a good treatment option for vascular anomalies of the head and neck region. Identification of accurate puncture sites due to multiplanar reconstruction of vascular and soft tissue maps leads to a successful and safe embolization procedure. No complications were reported in all cases even though a potential complication, namely efflux of the embolization toward the vascular pathway, might occur. To obtain a convincing minimal intervention that comes with high precision, a larger scale of clinical study needs to be conducted in the future because this study is presented with only a small number of subjects.

## Learning points

- CBCT-A provides good visualization and guidance for direct puncture embolization in vascular anomalies of the head and neck region by accurately identifying puncture sites through multiplanar reconstructions of vascular and soft tissue maps.
- CBCT-A and DSA combination as guidance for direct puncture helps to achieve successful embolization in otherwise difficult access and vascular anatomy when done through endovascular approach.
